# Impact of silencing hepatic SREBP-1 on insulin signaling

**DOI:** 10.1371/journal.pone.0196704

**Published:** 2018-05-03

**Authors:** Victoria Jideonwo, Yongyong Hou, Miwon Ahn, Sneha Surendran, Núria Morral

**Affiliations:** 1 Department of Medical and Molecular Genetics, Indiana University School of Medicine, Indianapolis, Indiana, United States of America; 2 Department of Biochemistry and Molecular Biology, Indiana University School of Medicine, Indianapolis, Indiana, United States of America; University of Cordoba, SPAIN

## Abstract

Sterol Regulatory Element Binding Protein-1 (SREBP-1) is a conserved transcription factor of the basic helix-loop-helix leucine zipper family (bHLH-Zip) that plays a central role in regulating expression of genes of carbohydrate and fatty acid metabolism in the liver. SREBP-1 activity is essential for the control of insulin-induced anabolic processes during the fed state. In addition, SREBP-1 regulates expression of key molecules in the insulin signaling pathway, including insulin receptor substrate 2 (IRS2) and a subunit of the phosphatidylinositol 3-kinase (PI3K) complex, PIK3R3, suggesting that feedback mechanisms exist between SREBP-1 and this pathway. Nevertheless, the overall contribution of SREBP-1 activity to maintain insulin signal transduction is unknown. Furthermore, Akt is a known activator of mTORC1, a sensor of energy availability that plays a fundamental role in metabolism, cellular growth and survival. We have silenced SREBP-1 and explored the impact on insulin signaling and mTOR in mice under fed, fasted and refed conditions. No alterations in circulating levels of insulin were observed. The studies revealed that depletion of SREBP-1 had no impact on IRS1^Y612^, Akt^S473^, and downstream effectors GSK3α^S21^ and FoxO1^S256^ during the fed state. Nevertheless, reduced levels of these molecules were observed under fasting conditions. These effects were not associated with changes in phosphorylation of mTOR. Overall, our data indicate that the contribution of SREBP-1 to maintain insulin signal transduction in liver is modest.

## Introduction

A complex network of molecules senses nutrient availability, activating anabolic processes during periods of abundance, and shutting down biosynthetic programs that consume energy when nutrients are scarce. In the liver, insulin signaling plays a pivotal role at promoting anabolic responses during fed conditions, including synthesis of glycogen and fatty acids from glucose, as well as inhibiting hepatic glucose production. Insulin binding to the insulin receptor/insulin-like growth factor (IGF-1) receptor elicits phosphorylation of insulin receptor substrates 1 and 2 (IRS1/2) on tyrosine residues, sending a downstream signal that activates phosphatidylinositol 3-kinase (PI3K) [1]. A main outcome of PI3K activation is phosphorylation of protein kinase B (Akt) [1]. Akt activation prompts the phosphorylation and nuclear exclusion of forkhead box O1 (FKHR or FoxO1), a key transcription factor that activates expression of gluconeogenesis genes. FoxO1 phosphorylation by Akt is a fundamental process to downregulate gluconeogenesis gene expression during the fed state. In addition, Akt inactivates glycogen synthase kinase 3 (GSK3), thereby activating the enzyme glycogen synthase and promoting glycogen biosynthesis [2]. Conversely, low insulin levels during fasting conditions lead to activation of the gluconeogenesis and glycogenolysis pathways, increasing hepatic glucose production.

Insulin signaling and Akt activity are interconnected with the mechanistic target of rapamycin (mTOR) pathway. TOR is a highly conserved protein from yeast to mammals, and plays a key role at orchestrating fundamental aspects of metabolism, cellular growth, proliferation and survival. Two complexes exist, TORC1 and TORC2, with distinct functions. In mammals, mTORC1 regulates ribosome biogenesis and protein synthesis [3], while mTORC2 regulates actin cytoskeleton organization [4, 5]. Unique protein subunits participate in the specific functions of each complex. The major partner of mTORC1 is regulatory-associated protein of mTOR (Raptor) [6, 7]. mTORC2 associates with rapamycin-insensitive companion of mTOR (Rictor) [5]. Insulin activates mTORC1 through Akt-mediated inhibition of GTPase-activating protein heterodimer tuberous sclerosis 1/2 (TSC1/2), an inhibitor of mTORC1 [8]. In addition, Akt directly phosphorylates mTOR at serine 2448 [8, 9]. Insulin also activates mTORC2, which in turn, phosphorylates Akt at serine 473 residue, a necessary step for full activation of Akt [10].

Importantly, insulin-induced mTORC1 activity upregulates expression of Sterol Regulatory Element Binding Protein-1 (SREBP-1) [11–14], a conserved transcription factor of the basic helix-loop-helix leucine zipper family (bHLH-Zip) that primarily controls expression of glycolysis and *de novo* lipogenesis (DNL) enzymes. SREBP-1a and SREBP-1c are isoforms of the same gene, and both regulate L-pyruvate kinase, acetyl-CoA carboxylase, fatty acid synthase, stearoyl-CoA desaturase 1, and mitochondrial glycerol-3-phosphate acyltransferase 1, among other genes in the lipogenesis and glycolysis pathways [15]. SREBPs are synthesized as precursors that are bound to the endoplasmic reticulum membrane. In response to specific signals, SREBPs transition to the Golgi, where they are cleaved, releasing the mature form, which translocates to the nucleus and activates expression of target genes [15]. mTORC1 activity is necessary for activation of SREBP-1 gene expression and for its processing from precursor to the mature form [11–14]. Thus, activation of SREBP-1 is a critical function of the Akt/mTORC1 signaling axis. Actually, multiple studies have provided evidence that SREBP-1 coordinates a variety of responses needed for cell survival and growth, including lipogenesis [15, 16]; glycogen synthesis [17, 18]; phagocytosis and membrane biosynthesis [19]; as well as insulin signaling molecules [20, 21]. Despite evidence indicating that SREBP-1 regulates IRS2 and PIK3R3 [20, 21], the overall contribution of SREBP-1 activity on hepatic insulin signaling is unknown. Here, we explored the impact of knocking-down SREBP-1 on the insulin signaling pathway and mTOR.

## Materials and methods

### Animals

All animal studies were in accordance with the National Institutes of Health guidelines and were approved by the Indiana University School of Medicine Institutional Animal Care and Use Committee. Male eight-week old C57BLKS/J mice were obtained from The Jackson Laboratory (Bar Harbor, ME), and allowed to acclimate for at least a week before experimentation. A standard 12 h light/12 h dark cycle (7 AM/7 PM) was maintained throughout the experiments. Mice were maintained in a BSL2-certified room and were fed rodent chow *ad libitum* and allowed free access of water. Mice (n = 5–6) were given 1x10^11^ viral particles (vp) by tail vein injection, and euthanized 8 days after adenovirus vector administration under *ad libitum* fed conditions, 24-h fasted or 24-h fasted followed by a 4.5-h refeeding period. Tissues were collected and snap frozen in liquid nitrogen and kept at -80°C.

Male C57BL/6J mice (The Jackson Laboratory) were used for isolation of primary hepatocytes.

### Adenoviral vector production

Helper-dependent or ‘gutless’ adenoviral (HD-Ad) vectors are the most advanced type of adenoviral vector, and are devoid of viral coding sequences, only retaining the inverted terminal repeats and packaging signal. The lack of viral genes virtually eliminates inflammatory responses and toxicity in mice and non-human primates [22–24]. Helper-dependent adenoviral vectors have identical tropism to first generation (E1-deleted) adenoviral vectors, and predominantly transduce the liver [22, 25]. HD-Ad vectors were generated in HEK293Cre cells, using a Cre-loxP system developed by Merck Laboratories and Microbix (Toronto, Canada) [26, 27]. The production of HD-Ad vectors expressing an shRNA to target SREBP-1 or a scrambled sequenced has been previously described [28]. After production, vectors were stored at -80°C in 10 mM Tris-HCl (pH 7.5), 1 mM MgCl_2_, 150 mM NaCl, 10% glycerol. Total particle counts were determined spectrophotometrically, as described [28].

For overexpression of SREBP-1c in primary hepatocytes, a first generation (E1-deleted) vector expressing the N-terminal form (amino acids 1~436) of human SREBP-1c was used (Eton Bioscience, San Diego, CA). An adenovirus without expression cassette (Null) was used as control. Both adenoviral vectors were grown in HEK293 cells [29] and stored as described for helper-dependent adenoviral vectors. Titers were determined by plaque assay in HEK293 cells.

### Primary hepatocyte isolation and culture

Primary hepatocytes were isolated from C57BL/6J mice using a two-step collagenase procedure followed by Percoll gradient centrifugation to separate hepatocytes from non-parenchymal cells, as previously described [30, 31]. Cell viability was assessed by trypan blue staining exclusion (>80% viability). Cells were seeded at a density of 4-6x10^5^ cells per well in 6-well plates, and incubated in a humidified 5% CO_2_ incubator at 37°C. Cells were allowed to attach for 4 hours, and medium was then replaced with fresh medium.

To address the impact of overexpressing SREBP-1c on insulin signaling, primary hepatocytes were infected with an adenovirus expressing human SREBP-1c or a control vector at MOI 20, 40, 60, 80 or 100. Medium was changed the next day. Cells were cultured in DMEM containing with 5 mM glucose, 10% FBS and 100 IU/ml penicillin/100 μg/ml streptomycin, 100 nM dexamethasone, and washed twice with 1x PBS, prior to harvesting.

### Western blotting

Liver tissue or primary hepatocytes were lysed in RIPA buffer (Thermo Scientific, Rockford, IL) containing protease and phosphatase inhibitors (Roche, Indianapolis, IN). Protein concentration was determined using the BCA kit from Pierce (Rockford, IL). Proteins (7–30 μg) were separated in 10% or 4–20% Tris-HCl SDS PAGE Criterion gel (Bio-Rad, Hercules, CA) and transferred to 0.2-mm PVDF membrane (Bio-Rad). Membranes were blocked with 5% BSA-TBST or 5% dry milk-TBST for 1–2 h and incubated with the following antibodies: ACACA/B (aka ACC1/2), IRS1, PDK-1^S241^, PDK-1, Akt^S473^, Akt (Pan), Foxo1^S256^, Foxo1, GSK3α^S21^, GSK3β^S9^, mTOR^S2448^, mTOR, Rictor, Raptor (Cell Signaling, Danvers, MA); α-tubulin, SREBP-1 MS-1207 (Thermo Scientific, Waltham, MA); β-actin, SREBP-1 H-160, glucokinase H-88 (Santa Cruz Biotechnology, Dallas, TX); IRS2 (EMD Millipore, Billerica, Massachusetts); IRS1^Y612^, GSK3 (Invitrogen, Life Technologies). HRP-conjugated secondary antibody was added and incubated at room temperature for 1 hour. Blots were developed with Pierce ECL kit (Thermo Scientific) and exposed to enhanced chemiluminescence (ECL) film (GE Healthcare, Piscataway, NJ). Bands on blots were quantified by densitometry using ImageJ v1.48s, and results were normalized to control protein, as specified in the figure legends.

### Serum chemistries

Blood glucose was measured with an Ascensia Elite XL meter (Bayer, Tarrytown, NY), from a drop collected from the tail vein. β-hydroxybutyrate was analyzed with a kit from Pointe Scientific (Canton, MI). Insulin was analyzed by the Translation Core of the Center for Diabetes and Metabolic Diseases using a Rodent Insulin Chemiluminescence ELISA assay (ALPCO, Salem, NH). All reactions were carried out in duplicate, following the manufacturer’s instructions.

### Statistical analysis

Numerical values represent mean ± SD. *P* values were calculated using unpaired two-tailed Student’s *t*-tests. A *P* value of less than 0.05 was considered statistically significant.

## Results and discussion

### Silencing SREBP-1 decreases insulin signaling in mouse liver

**Fig 1:**
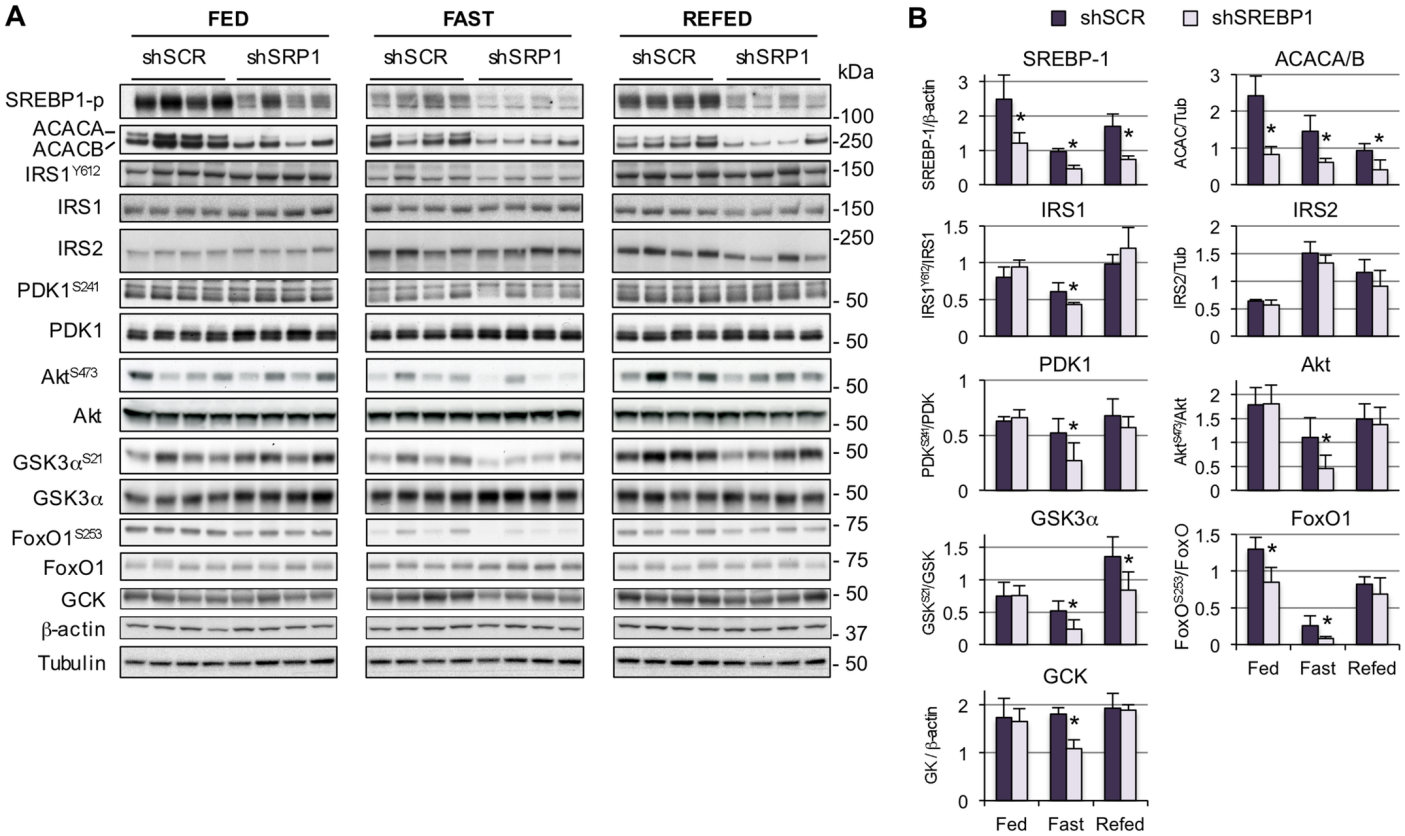
Silencing SREBP-1 *in vivo* reduces hepatic insulin signaling. Mice were administered 1x10^11^ viral particles of HD-Ad.shSREBP1 or HD-Ad.shSCR, and euthanized after 8 days under fed, 24-hour fasted or 24-hour fasted followed by a 4.5 hour refeeding period. **(A)** Tissue lysates were subjected to immunoblotting analysis using the antibodies shown on the left. **(B)** Densitometry analysis of insulin signaling molecules. Values represent mean ± SD (n = 4); *p<0.05 shSCR vs shSREBP1.

In addition to its role in controlling expression of genes of glycolysis and fatty acid metabolism [15, 18], SREBP-1 inhibits IRS2 [21] and activates expression of a subunit of the PI3 kinase complex, phosphatidylinositol-3 kinase regulatory subunit p55γ (PIK3R3) [20], two essential molecules in the insulin signaling pathway. To investigate the relative contribution of SREBP-1 to regulate hepatic insulin signaling, mice received a helper-dependent adenoviral vector expressing a short hairpin RNA (shRNA) to knock-down SREBP-1, or a control vector expressing a scrambled sequence. Mice were studied a week later under fed conditions, 24-hour fasted, or 24-hour fasted followed by a 4.5-hour refeeding period. SREBP-1 silencing did not significantly affect body weight or insulin levels relative to the shSCR control group (Table 1). Blood glucose was only slightly decreased under fasting conditions (Table 1). As expected, ACACA and ACACB were decreased, confirming that silencing resulted in decreased SREBP-1 target gene expression. Remarkably, loss of SREBP-1 did not lead to changes in IRS2, contrary to what would have been anticipated, based on the known inhibitory effects on expression of this molecule [21]. SREBP-1 deficiency did not affect insulin signaling under the fed state, when insulin levels are high (Fig 1). However, a reduction in the levels of IRS1^Y612^ phosphorylation was observed under fasting conditions (i.e., low insulin), and a decrease was also evident in downstream molecules, including PDK-1^S241^, Akt^S473^, GSK3α^S21^, and FoxO1^S256^, suggesting that depletion of SREBP-1 had a negative impact on this pathway. Nevertheless, the decrease was moderate and was distinct only under fasting conditions, suggesting that nutrients and/or high insulin attained under fed conditions are sufficient to maintain normal insulin signal transduction. Interestingly, SREBP-1 deficiency resulted in changes in glucokinase expression following the same trends (i.e. lower under the fasted, but not the fed state). This indicates that, similar to IRS2, SREBP-1 is permissive for glucokinase expression, and other factors may be more relevant for its regulation. Even though some studies point at SREBP-1 as the mediator of insulin-induced glucokinase expression, other studies have argued against a major role for this transcription factor [32]. Indeed, multiple transcription factors have been shown to directly activate glucokinase gene expression, including Kruppel-like factor 6 [33], peroxisome proliferator-activated receptor gamma (PPARγ) [34], liver receptor homolog 1 (LRH-1) [35], hepatocyte nuclear factor 4 (HNF4) [36], and hypoxia-inducible factor 1 alpha (HIF1α) [36]. In addition, glucokinase protein is stabilized by other proteins, including glucokinase regulatory protein (GKRP), Bcl-2-associated agonist of cell death (BAD), and 6-phosphofructo-2-kinase/fructose 2,6-bisphosphatase (PFK2/FBP2), in response to nutritional signals [37]. Thus, overall levels of glucokinase in liver may be influenced by multiple factors at the transcriptional and post-translational level.

**Table 1:** Body weight, blood glucose and serum insulin levels in mice.

	FED		FASTED		EFED	
	shSCR	shSREBP	shSCR	shSREBP	shSCR	shSREBP
*Body weight (g)*	25.0±1.2	26.2±1.3	21.8±1.1	22.4±2.1	26.7±2.0	23.0±1.7
*Blood glucose (mg/dL)*	106.6±10.9	106.2±9.8	41.0±3.1	36.4±3.1*	133.7±14.0	148.0±10.8
*Insulin (ng/ml)*	0.65±0.2	0.84±0.3	0.46±0.1	0.39±0.1	1.42±0.5	1.30±0.6

*p = 0.047 relative to shSCR; n = 5–6

**Fig 2:**
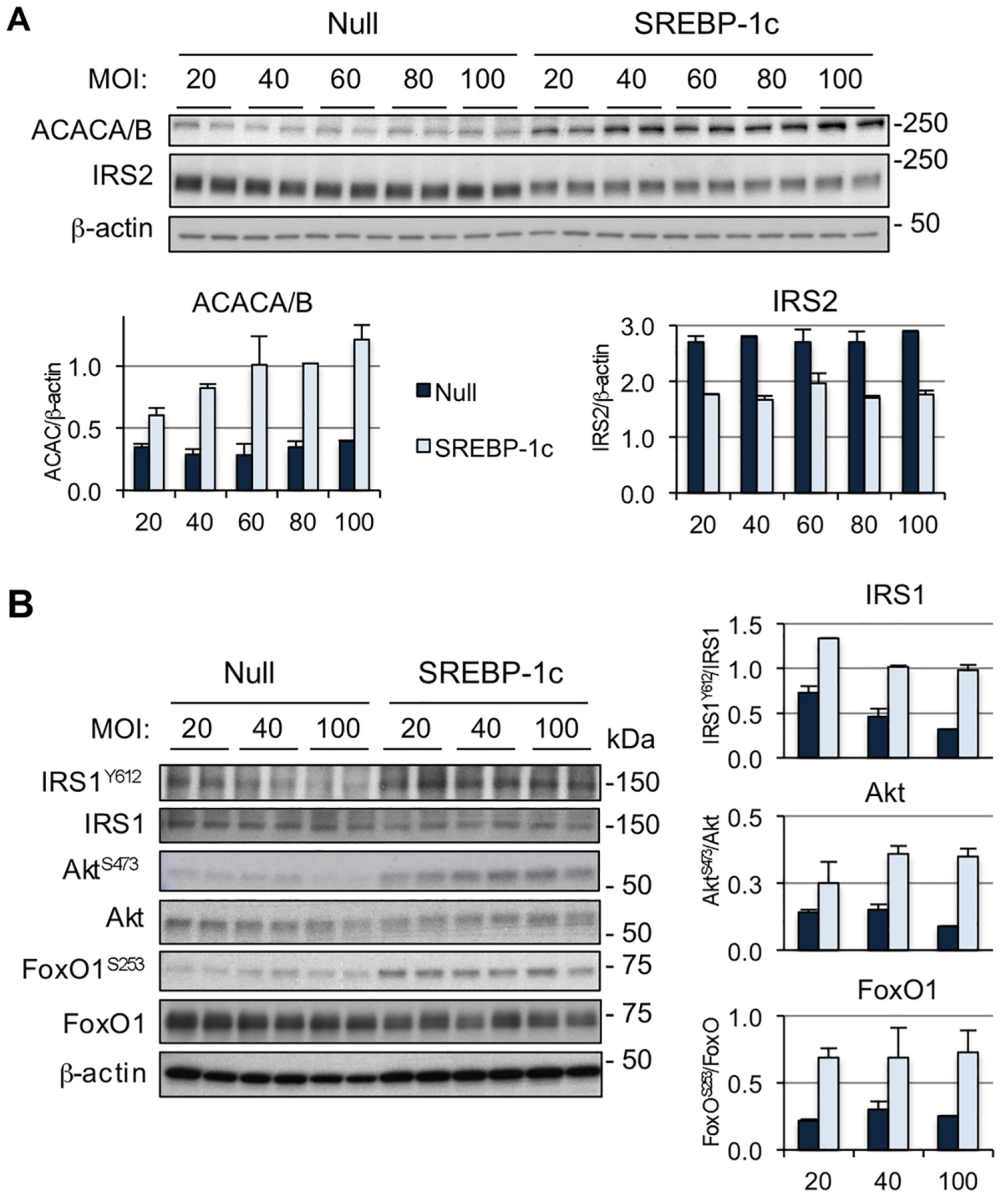
SREBP-1 expression increases insulin signaling in primary hepatocytes. **(A)** Mouse primary hepatocytes were cultured in DMEM containing 5 mM glucose, 10% FBS and 100 IU/ml penicillin/100 μg/ml streptomycin, 100 nM dexamethasone. Cells were transduced with an adenovirus expressing SREBP-1c or a control vector (Null) at the multiplicity of infection (MOI) indicated on the top. Cells were harvested 48 hours later. IRS2 levels decreased while ACACA/B increased in hepatocytes treated with SREBP-1c, as expected. **(B)** Insulin signaling in primary hepatocytes transduced with an adenovirus expressing the mature form of human SREBP-1c or a control vector (Null) at multiplicity of infection (MOI) 20, 40, or 100. Data are representative of 2 separate experiments.

To further evaluate the influence of SREBP-1 activity on insulin signaling, primary hepatocytes were transduced with an adenoviral vector expressing the mature form of human SREBP-1c or with a control vector, at multiplicity of infection (MOI) 20, 40, 60, 80 and 100. As expected, SREBP-1 targets IRS2 and ACACA/B (aka ACC1/2) were downregulated and upregulated, respectively (Fig 2A). Overexpression of SREBP-1c resulted in opposite effects to those observed by silencing SREBP-1 *in vivo*, i.e., increased IRS1^Y612^ and Akt^S473^ phosphorylation, as well as the downstream Akt target FoxO1^S256^ (Fig 2B and S1 Fig). These data support the concept that, in hepatocytes, short-term overexpression of SREBP-1 is associated with enhanced insulin signal transduction, and are in agreement with recent data in human hepatocellular carcinoma (HCC). Overexpressing SREBP-1 in HCC cell lines, accelerated their growth and reduced apoptosis. Remarkably, this was accompanied by increased levels of phosphorylated Akt^S473^ [38]. Conversely, in HCC cell lines expressing high SREBP-1 levels, knocking down SREBP-1 reduced Akt^S473^ activity [38]. Of note, chronic SREBP-1 overexpression in liver has been associated with hepatic steatosis and insulin resistance [39, 40], due to accumulation of lipid molecules that interfere with the insulin signaling pathway [41, 42]. Thus, increased SREBP-1 activity may be perceived at the short-term as a signal of nutrient abundance and cell growth, but its prolonged overexpression leads to negative effects due to buildup of lipid classes that hinder insulin signaling.

Glucokinase protein levels were not affected by overexpression of SREBP-1 in primary hepatocytes (S2 Fig). This could be due to the fact that primary hepatocytes do not exhibit the same expression profiles of the intact liver, and transcription factors/cofactors that are involved in the insulin-induced response, may not be present in the primary hepatocytes. Alternatively, it could be due to changes in levels of glucose metabolites, known to downregulate glucokinase expression [37].

Altogether, the data *in vivo* and *in vitro* suggest that SREBP-1 influences insulin signaling, although only to a moderate extent. Short-term (1 week) SREBP-1 depletion was associated with lower IRS1^Y612^, Akt^S473^ and downstream molecules, GSK3α^S21^ and FoxO1^S256^, under fasting conditions. Thus, in the fed/refed state, nutrients (e.g., fatty acids, cholesterol) and/or high insulin, are sufficient to restore normal signaling. Remarkably, loss of SREBP-1 did not have consequences for IRS2, even though its overexpression in primary hepatocytes resulted in decreased IRS2. Our data suggest that other factors may be more important at maintaining basal IRS2 levels than SREBP-1. Contrary to a previous report [21], expressing SREBP-1 in primary hepatocytes had no effect on total levels of IRS1. Furthermore, IRS1 did not change upon SREBP-1 depletion. Thus, it is possible that the alterations in Akt^S473^ activity and downstream Akt effectors such as GSK3α and FoxO1, were mostly influenced by IRS1 activity, and not IRS2.

Although the molecular mechanisms guiding the changes in insulin signal transduction are unknown, it is possible that multiple intracellular factors are coordinated to sense energy availability and elicit this response. First, it has been shown that silencing SREBPs triggers changes in the composition of lipid rafts [43]. Lipid rafts are membrane microdomains with unique lipid composition, and are essential for numerous cellular functions, including signaling events [44]. Lipid rafts are rich in sphingolipids and cholesterol phospholipids. Saturated fatty acids are the main components of the side chains of phospholipids [44], and SREBP-1 plays a central role in their synthesis. Indeed, the PI3K-Akt-mTORC1 axis is vital to upregulate SREBP-1 and 2 to attain appropriate cellular levels of fatty acids and cholesterol, as well as for the integrity of lipid rafts [43]. Furthermore, it has been shown that the ratio of monounsaturated to saturated fat in total lipids is critical for Akt signaling and Akt^S473^ phosphorylation [45]. In addition, changes in the rate of glycolysis and lipogenesis (whose genes are regulated by SREBP-1) are likely to be detected by energy sensors like AMPK [46], a master regulator of energy homeostasis, leading to changes in insulin signaling to control overall cellular homeostasis.

### Silencing SREBP-1 is not associated with decreased mTOR activity

**Fig 3:**
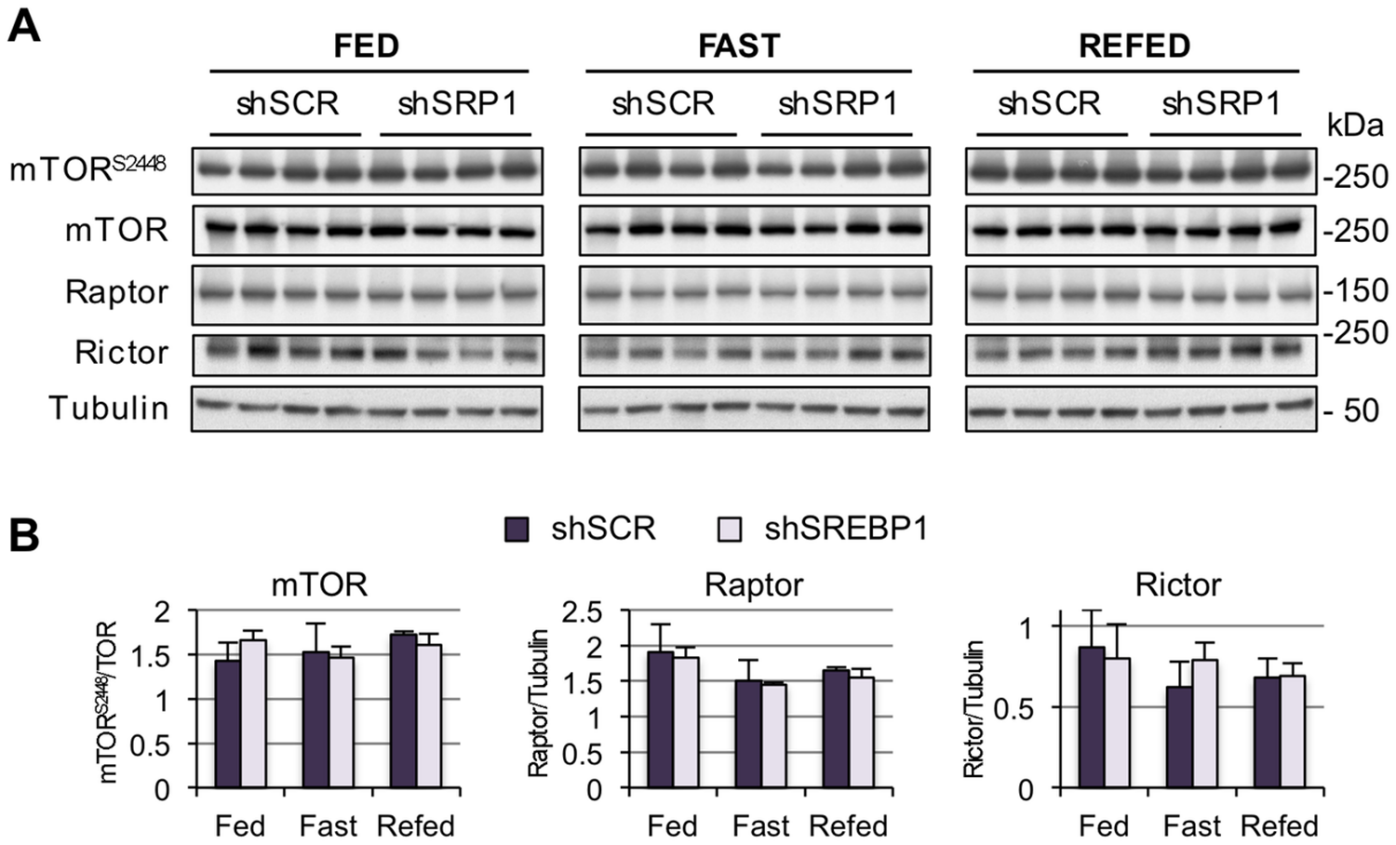
SREBP-1 depletion has no impact on mTOR. **(A)** Mice were treated as described in Fig 1. Tissue lysates were subjected to immunoblotting analysis using the antibodies shown on the left. **(B)** Densitometry analysis of insulin signaling molecules. Values represent mean ± SD (n = 4).

The mTOR pathway functions as the hub for sensing nutrient abundance and changes in energy supplies [47]. The Akt/mTORC1 axis has emerged as a critical regulatory point in the control of cell growth and cellular proliferation, and both molecules are targets for drug development in cancer treatment [48–52]. Two main mechanisms activate mTOR in response to insulin signaling, both mediated by Akt: (i) direct phosphorylation of mTOR at residue 2448 [8, 9]; (ii) phosphorylation of the tuberous sclerosis complex 2 (TSC2), thereby activating mTORC1 [53]. Given the dependence of mTOR on Akt activity, and that loss of SREBP-1 activity reduced Akt phosphorylation under fasting conditions, we questioned whether the mTOR pathway would be affected. Depleting SREBP-1 *in vivo* did not induce changes in mTOR phosphorylation at S2448, or in total levels of mTOR and the subunits of mTORC1 and mTORC2, Rictor and Raptor (Fig 3). Thus, the lower insulin sensitivity resulting from silencing SREBP-1 did not have an impact on mTOR. It is possible that the moderate decrease in insulin signal transduction during fasting was not sufficient to influence mTOR. Alternatively, mTOR activity is most relevant during nutrient abundance (fed state) [54], and insulin signaling was not affected in SREBP-1-depleted animals.

## Conclusion

SREBP-1 is a transcription factor that controls important aspects of hepatic function, including carbohydrate and lipid metabolism, in response to insulin [15, 17, 18]. SREBP-1 positively correlates with mTOR activity, and both are upregulated in animal models of type 2 diabetes and in cancer [11, 40, 55–59]. The existing evidence that SREBP-1 controls expression of molecules in the insulin signaling pathway, including IRS2 and PIK3R3 [20, 21], suggests that feedback mechanisms exist between SREBP-1 and this pathway. Our data indicates that SREBP-1 activity is dispensable for normal insulin signal transduction under fed conditions. Depleting SREBP-1 in the fasted state results in a modest decrease in insulin signaling, without influencing mTOR activity. Even though the specific molecular event/s leading to this decrease remain to be determined, it is possible that they are linked to a reduction in metabolites generated in the *de novo* lipogenesis pathway. The presence of these metabolites in the diet and/or the higher level of insulin during the fed state are sufficient to maintain normal signal transduction levels. Overall, the contribution of SREBP-1 to sustain insulin signaling is modest.

## Supporting information

S1 Fig SREBP-1c expression increases insulin signaling. (PDF)

S2 Fig Glucokinase is not upregulated in primary hepatocytes overexpressing SREBP-1c. (PDF)
