# Supplementary material for: Impact of silencing hepatic SREBP-1 on insulin signaling
Source: PLoS One. 2018 May 3;13(5):e0196704. doi: 10.1371/journal.pone.0196704 (PMC5933792; doi:10.1371/journal.pone.0196704)
Supplement: S1 Fig — (PDF) [file pone.0196704.s001.pdf]

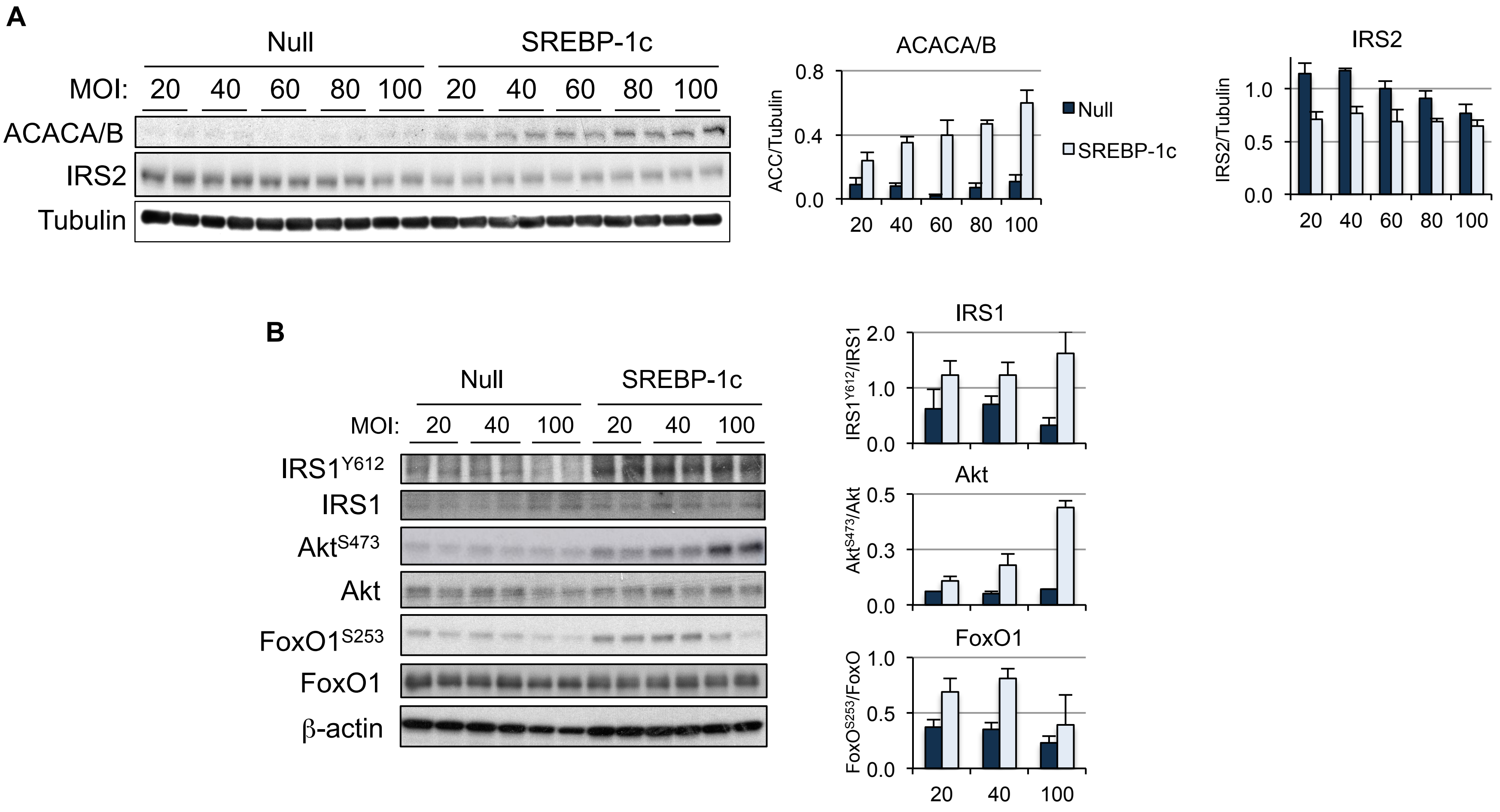

S1 Fig. SREBP-1c expression increases insulin signaling. (A) Primary hepatocytes were cultured in DMEM containing with 5 mM glucose, 10% FBS, 100 IU/ml penicillin/100 µg/ml streptomycin, and 100 nM dexamethasone. Cells were transduced with an adenovirus expressing SREBP-1c or a control vector (Null) at the multiplicity of infection (MOI) indicated on the top. Cells were harvested 72 hours later. **(B)** Analysis of molecules of the insulin signaling pathway.
