# Supplementary material for: Impact of silencing hepatic SREBP-1 on insulin signaling
Source: PLoS One. 2018 May 3;13(5):e0196704. doi: 10.1371/journal.pone.0196704 (PMC5933792; doi:10.1371/journal.pone.0196704)
Supplement: S2 Fig — (PDF) [file pone.0196704.s002.pdf]

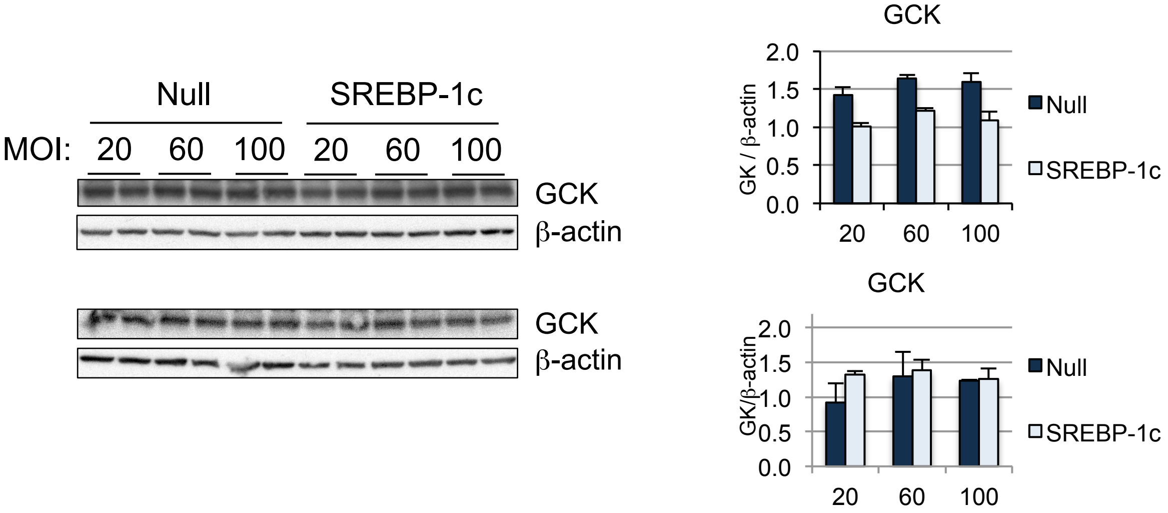

S2 Fig. Glucokinase is not upregulated in primary hepatocytes overexpressing SREBP-1c. Primary hepatocytes were cultured as described in Supplementary Fig.1. Cells were transduced with an adenovirus expressing SREBP-1c or a control vector (Null) at the multiplicity of infection (MOI) indicated on the top. Cells were harvested 48 hours later. Two independent experiments are shown. GCK, glucokinase.
